# Exploring Key Genes to Construct a Diagnosis Model of Dilated Cardiomyopathy

**DOI:** 10.3389/fcvm.2022.865096

**Published:** 2022-04-27

**Authors:** Youyang Zheng, Zaoqu Liu, Xinyue Yang, Siyuan Weng, Hui Xu, Chunguang Guo, Zhe Xing, Long Liu, Libo Wang, Qin Dang, Chunguang Qiu

**Affiliations:** ^1^Department of Cardiovascular Medicine, The First Affiliated Hospital of Zhengzhou University, Zhengzhou, China; ^2^Department of Interventional Radiology, The First Affiliated Hospital of Zhengzhou University, Zhengzhou, China; ^3^Interventional Institute of Zhengzhou University, Zhengzhou, China; ^4^Interventional Treatment and Clinical Research Center of Henan Province, Zhengzhou, China; ^5^Department of Endovascular Surgery, The First Affiliated Hospital of Zhengzhou University, Zhengzhou, China; ^6^Department of Neurosurgery, The Fifth Affiliated Hospital of Zhengzhou University, Zhengzhou, China; ^7^Department of Hepatobiliary and Pancreatic Surgery, The First Affiliated Hospital of Zhengzhou University, Zhengzhou, China; ^8^Department of Colorectal Surgery, The First Affiliated Hospital of Zhengzhou University, Zhengzhou, China

**Keywords:** dilated cardiomyopathy, functional analysis, machine learning, diagnostic model, immune infiltration

## Abstract

**Background:**

Dilated cardiomyopathy (DCM) is characterized by left ventricular dilatation and systolic dysfunction. The pathogenesis and etiologies of DCM remain elusive. This study aims to identify the key genes to construct a genetic diagnosis model of DCM.

**Methods:**

A total of 257 DCM samples from five independent cohorts were enrolled. The Weighted Gene Co-Expression Network Analysis (WGCNA) was performed to identify the key modules associated with DCM. The latent mechanisms and protein-protein interaction network underlying the key modules were further revealed. Subsequently, we developed and validated a LASSO diagnostic model in five independent cohorts.

**Results:**

Two key modules were identified using WGCNA. Novel mechanisms related to the extracellular, mitochondrial matrix or IL-17 signaling pathway were pinpointed, which might significantly influence DCM. Besides, 23 key genes were screened out by combining WGCNA and differential expression analysis. Based on the key genes, a genetic diagnosis model was constructed and validated using five cohorts with excellent AUCs (0.975, 0.954, 0.722, 0.850, 0.988). Finally, significant differences in immune infiltration were observed between the two groups divided by the diagnostic model.

**Conclusion:**

Our study revealed several novel pathways and key genes to provide potential targets and biomarkers for DCM treatment. A key genes’ diagnosis model was built to offer a new tool for diagnosing DCM.

## Introduction

Dilated cardiomyopathy (DCM) is defined by left ventricular dilatation and systolic dysfunction in the absence of known abnormal loading conditions or significant coronary artery disease (1). DCM patients usually have a progressively exacerbated condition and poor prognosis. Deaths could exist in any stage of DCM. The prevalence of DCM was > 1 per 250 individuals (2). Moreover, the prevalence of cardiomyopathy had risen by 27% in just 10 years, according to the Global Burden of Disease study in 2015. The last decades have seen large advances in our understanding of cardiomyopathy. DCM could be classified as genetic, mixed, or acquired forms (3). Mutations in genes that encode cytoskeleton, sarcomere, transcriptional pathways, nuclear envelope, and mitochondrial proteins are genetic causes of DCM. Etiologies of acquired DCM are various, including infections, autoimmunity, toxins, and endocrine disorders (4). However, numerous cases are always categorized as idiopathic DCM because of limited diagnostic conditions, which leads to symptomatic rather than specific treatment (5). To date, the genetic factors and pathogenesis of DCM are still not fully understood. Due to unclear etiologies, there were few specific treatments of DCM. Therefore, further studies need to be carried out to investigate the potential mechanisms of DCM. Also, accurate diagnostic approaches are urgently needed.

Previously, most of the common pathogenic genes of DCM were identified from basic research, such as TTN and LMNA (6, 7). Few studies used bioinformatic methods to explore potential genes of DCM. In recent years, high-throughput sequencing technologies accelerated the development of medical studies, offering a powerful tool to detect possible gene mutations of diseases. Machine-learning developed rapidly and is widely used in medical research artificial intelligence (AI), which is usually used for dimensionality reduction. Based on that, our study proposed a genetic diagnosis model of DCM, which might exert distinct influences for clinical diagnosis.

This study aims to identify underlying genes and construct an ideal genetic diagnosis model. The microarray data were collected from the Gene Expression Omnibus (GEO).^1^ Twenty-three key genes of DCM were screened out based on WGCNA and differential analysis, most of whom were never reported before. Besides, we pinpointed several mechanisms highly associated with DCM according to functional analysis of key genes. Furthermore, we built a genetic diagnosis model and validated it in four cohorts. Additionally, more analyses were conducted to explore DCM comprehensively in this study.

## Materials and Methods

### Dataset Collection and Preprocessing

Five datasets [GSE5406 (*n* = 102), GSE57338 (*n* = 218), GSE116250 (*n* = 51), GSE42955(*n* = 17), GSE19303(*n* = 48)] were selected out from Gene Expression Omnibus (GEO) database using keywords “dilated cardiomyopathy” or “DCM,” including 257 DCM samples and 179 controls. The screening criteria were as follows: First, the dataset must include the DCM cases and controls. Second, all samples should be derived from ventricular myocytes. Third, the number of samples should be greater than 10 to ensure the quality of WGCNA. Fourth, the raw or processed data should be available in the GEO database for subsequent analysis. Gene expression matrices of five datasets were extracted using the R. Then, a gene expression matrix of overlapped genes was obtained after taking the interaction of five datasets, which was the input file of WGCNA.

### Weighted Gene Co-expression Network Analysis

Weighted Gene Co-Expression Network Analysis (WGCNA) has become an effective tool to screen key genes with high biological significance, which inspired researchers to explore the mechanisms of diseases. To identify key modules highly correlated with DCM, WGCNA was performed with WGCNA package (8) in R. The expression of genes was ranked in descending order, calculated by the standard deviation (SD). Then, the top 5,000 genes were selected for further analysis. Moreover, we performed a hierarchical clustering analysis to exclude the outlier samples for the rationality of WGCNA. The Pearson correlations value between each gene pair was calculated to obtain a gene similarity matrix. Then, the formula, a_*ij*_ = | S_*ij*_ | ^β^ (a_*ij*_: adjacency matrix between gene i and j, S_*ij*_: similarity matrix of all gene pairs, β: the soft threshold) was used to construct adjacency matrix. The optimal β was selected to satisfy the scale-free distribution by the “pickSoftThreshold” function in the WGCNA package, making the correlations more distinguishable. Next, the adjacency matrix was transformed to topological overlap matrix (TOM) and 1—TOM, reflecting the similarity and dissimilarity among genes, respectively. Finally, we utilized the hierarchical clustering method to classify genes into different modules. The module eigengene (ME) was calculated, representing the gene expression profiles of each module. The modules that highly correlated with DCM were key modules for further analysis. A value of *P* < 0.05 was considered statistically significant. The settings of parameters were as follows. The soft threshold β = 9, minModuleSize = 50, mergeCutHeight = 0.3 and deepSplit = 2.

### Protein-Protein Interaction Network

Search Tool for Retrieval of Interacting Genes/Proteins (STRING version 11.0) is a database to construct a protein-protein interaction (PPI) network, including human proteins and their interactions (9). We built a PPI network of the key modules using the database. Subsequently, we visualized the network with Cytoscape software (version 3.9.0) (10). The confidence score was set to 0.4. Then, the cluster with most genes was extracted with Molecular Complex Detection (MCODE), a plug-in of Cytoscape, which can analyze the topological characteristics of the PPI network. The parameters of MCODE were all set using default settings.

### Gene Ontology and Pathway Analysis

The genes of key modules were subjected to functional enrichment analysis, including Gene Ontology (GO) and Kyoto Encyclopedia of Genes and Genomes (KEGG) pathway analysis. The clusterProfiler R package was used in these processes with an adjusted *P*-value < 0.05.

### Differential Expression Analysis

The three datasets (GSE5406, GSE57338, GSE116250) were utilized to identify differentially expressed genes (DEGs) with the “limma” R package. | log_2_(foldchange)| > 0.667 and the adjusted *p*-value < 0.05 were used as screening criteria. Furthermore, the up-regulated and down-regulated genes were taken interactions across three cohorts, respectively. Subsequently, to further determine highly positively correlated genes in key modules, intersections were taken again between the results of WGCNA and differential analysis.

### Construction and Validation of Diagnostic Model

The initial model construction was performed in GSE57338. Least absolute shrinkage and selection operator (LASSO) is an algorithm generally used in medical studies to select the best variables for the diagnostic model (11–13). The optimal λ was determined by setting cross-fold validation to 10. We used the “glmnet” (14) R package to perform LASSO, setting alpha to 1. Four datasets (GSE5406, GSE116250, GSE42955, GSE19303) were implemented as validation cohorts to verify the diagnostic performance. Receiver operating characteristics (ROC) were plotted to assess the diagnostic model. The LASSO model gave each sample a risk score for the diagnostic prediction. The median value of risk scores was employed as the criteria for grouping in GSEA.

### Gene Set Enrichment Analysis

To explore potential mechanisms closely associated with the risk of DCM, we classified the samples of the modeling dataset into high-risk and low-risk groups according to the median risk score. Next, the correlations were calculated between the risk score of each sample and the expression of each gene. Then, we ranked genes in descending order based on the correlations. Finally, Gene Set Enrichment Analysis (GSEA) was performed using two collections (c5.go.v7.4.symbols.gmt and c2.cp.kegg.v7.4.symbols.gmt) in Molecular Signatures Database. | NES| > 1.50, adjusted *P*-value < 0.01, and FDR < 0.01 were determined as cutoff criteria.

### Analysis of Immune Infiltration

The single-sample GSEA (ssGSEA) is an extension of GSEA, which can generate the enrichment score for an individual sample. ssGSEA is a widely used algorithm to calculate the abundance of various immune cells, pathways or functions based on the gene expression profiles of a given sample (15–17). To observe differences in immune infiltration between high-risk and low-risk groups, we used ssGSEA to calculate each sample’s abundance of infiltrating immune cells with GSVA v1.42.0 package in R (18). The differences between the two groups were visualized intuitively in different plots. Additionally, we calculated the correlation coefficients between the risk scores of samples and the abundance of immune cells to explore the primary immune cells that participate in the process of DCM.

### Statistical Analysis

Statistical analyses and plotting were conducted in R (version 4.0.5). Pearson’s correlations and Spearman’s correlation were performed to calculate correlations. The most valuable genes with non-zero coefficients were selected by LASSO logistic regression. Statistical significance was considered at *P* < 0.05.

## Results

### Data Collection

**FIGURE 1:**
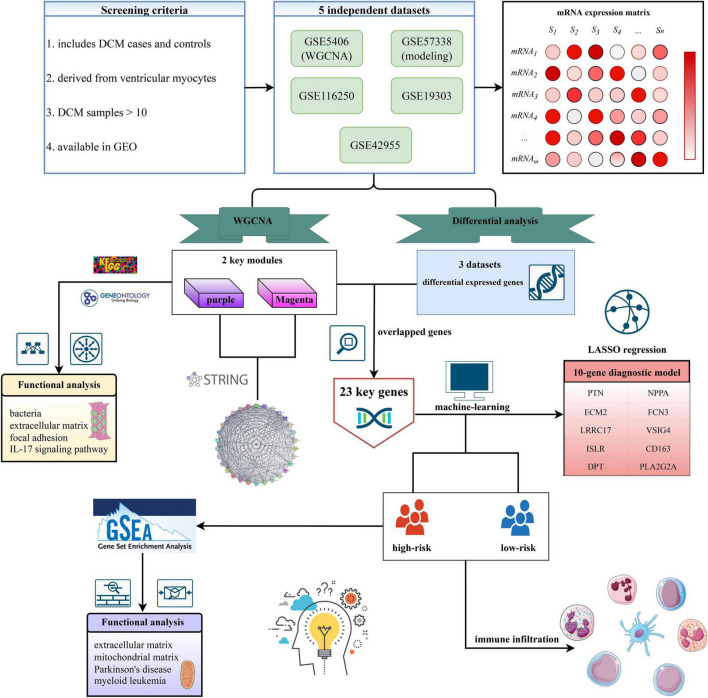
The workflow of this study.

Based on the criteria mentioned above, five independent datasets were selected from GEO. The details of these datasets were shown in Supplementary Table 1, including the basic information of datasets and functions in our study. The workflow of this study was shown in Figure 1.

### The Construction of Gene Co-expression Network

**FIGURE 2:**
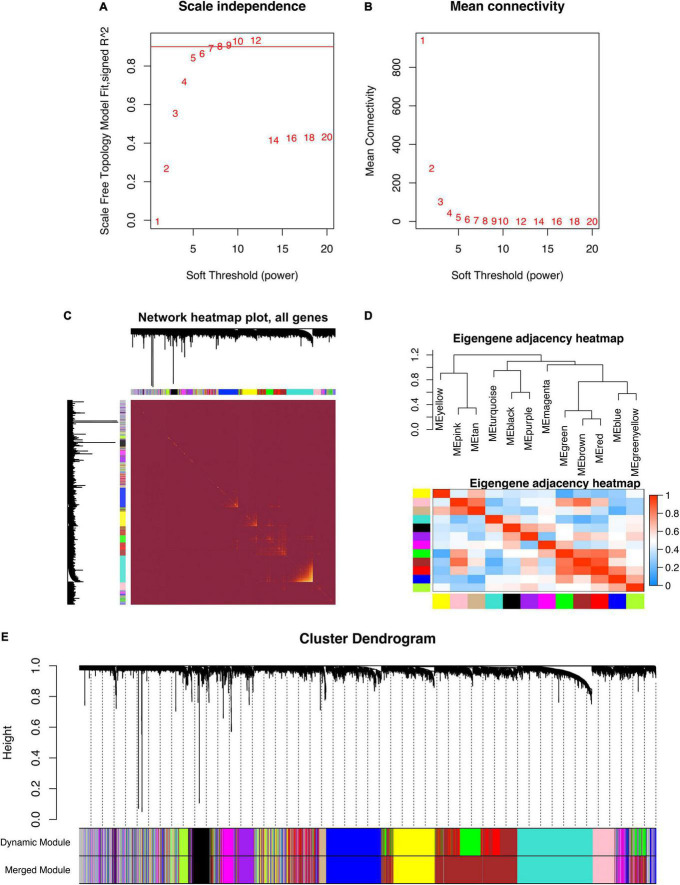
The construction of weighted gene co-expression network. **(A)** Scale-free topological indices at various soft-thresholding powers. **(B)** The correlation analysis between the soft-thresholding powers and mean connectivity of the network. **(C)** The heatmap of the topological overlap matrix of genes selected by WGCNA. **(D)** The heatmap of the eigengene adjacency. **(E)** Gene clustering diagram based on hierarchical clustering under optimal soft-thresholding power.

**FIGURE 3:**
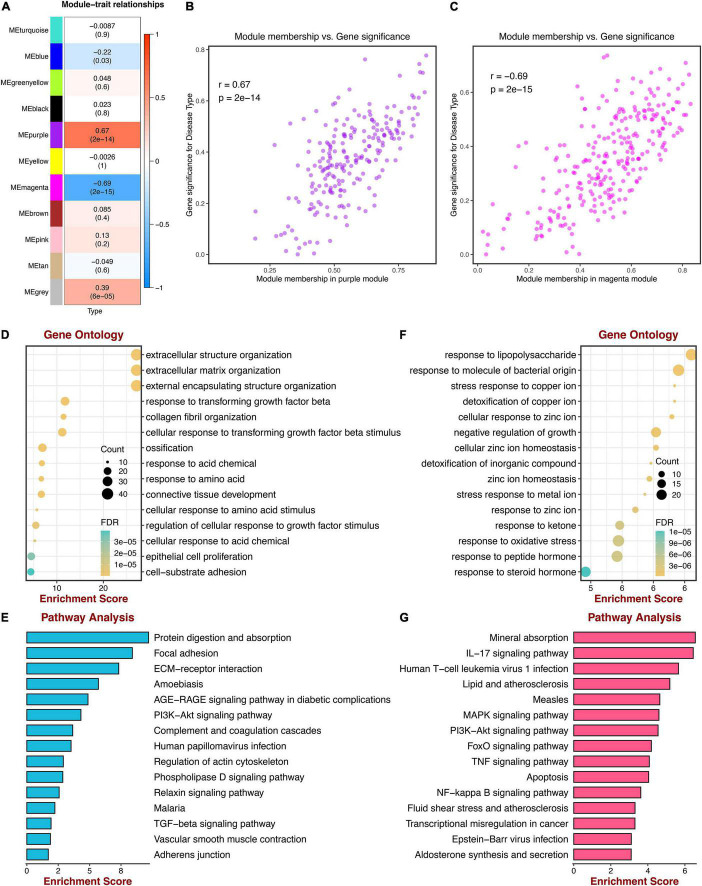
Correlations between gene modules and DCM; GO, and KEGG enrichment analysis. **(A)** Correlations between gene modules and DCM status. **(B)** The correlation between the purple module memberships and the gene significance for DCM. **(C)** The correlation between the magenta module memberships and the gene significance for DCM. **(D)** Go enrichment analysis of genes in the purple module. **(E)** KEGG pathway analysis of genes in the purple module. **(F)** GO enrichment analysis of genes in the magenta module. **(G)** KEGG pathway analysis of genes in the magenta module.

GSE5406 dataset was utilized as the training dataset of WGCNA. Before the network construction, we calculated the gene correlation matrix in order to meet the scale-free network for the biological hypothesis. The matrix was further converted to the adjacent matrix *via* the soft threshold β. As illustrated in Figures 2A,B, R2 achieved more than 0.9 when β = 9, which became the power of our adjacency matrix. To make the segmentation of modules easier, the topological overlap matrix (TOM) was transformed from the adjacency matrix and displayed in Figure 2C. Subsequently, 13 co-expression modules were identified using the hierarchical clustering method. An eigengene adjacency heatmap depicted the correlations between modules (Figure 2D). The eigengene represented the gene expression profile of each module. To simplify the network, we merged modules according to the similarity > 0.75 (Figure 2E). Ultimately, 11 modules were identified, two of which were highly relevant to DCM (Figure 3A). The purple module had the strongest positive correlation with DCM, including 221 genes. The magenta module was significantly negatively correlated with DCM, including 262 genes. The associations among genes, module membership, and the presence of disease were shown in Figures 3B,C.

### Functional Enrichment Analysis of Key Modules

To evaluate the functional enrichment of 2 key modules, we performed GO and KEGG pathway analysis. Genes of the purple module were significantly enriched in “extracellular matrix organization,” “extracellular structure organization,” “external encapsulating structure organization,” all of which were terms about extracellular matrix (ECM), as shown in Figure 3D. KEGG pathway terms were related to “Protein digestion and absorption,” “Focal adhesion,” and “ECM-receptor interaction,” which may play essential roles in DCM (Figure 3E). Meanwhile, the top 3 GO terms were enriched by genes of the magenta module, including “response to lipopolysaccharide,” “response to molecule of bacterial origin,” and “stress response to copper ion,” which were mainly associated with bacterial infection (Figure 3F). The KEGG pathways suggested that the IL-17 signaling pathway and lipid metabolism may be potential pathways of DCM (Figure 3G).

### The Hub Genes of Key Modules

**FIGURE 4:**
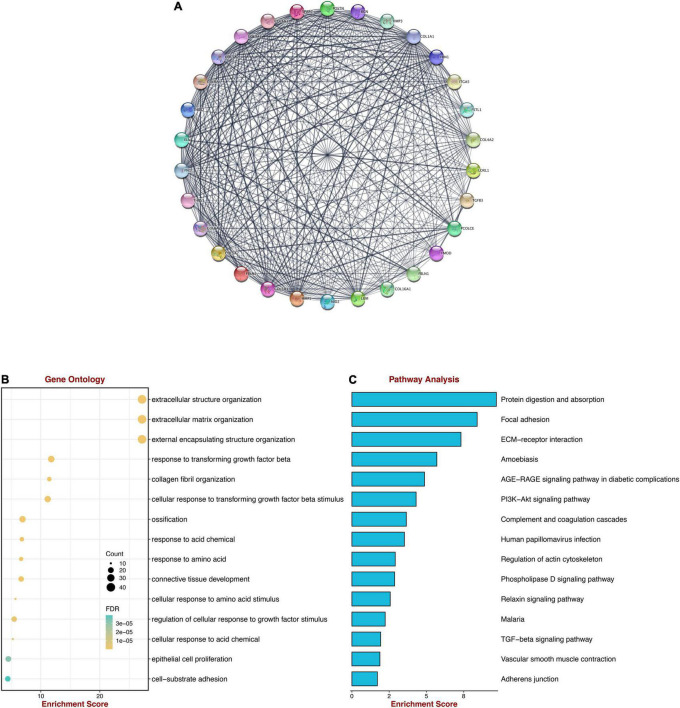
Protein-protein network and functional enrichment analysis of hub genes. **(A)** The protein-protein network of two modules. **(B)** GO enrichment analysis of the hub genes. **(C)** KEGG pathway analysis of the hub genes.

To seek the hub genes and pathways of two modules, the purple and magenta modules were combined to construct the PPI network and identify the hub genes using Cytoscape and MCODE. Consequently, the largest cluster consists of 30 hub genes such as BGN, COL1A1, COL1A2, FBLN1, FBLN2, THBS1, THBS2 etc., which symbolized genes of two modules to some extent (Figure 4A). To validate the importance of these hub genes, we also performed GO and KEGG pathway analysis (Figures 4B,C). The result was highly like the purple module’s functional enrichment such as “Extracellular matrix organization,” and “Focal adhesion,” either GO or KEGG pathway. Hence, these similar GO terms or KEGG pathways may have crucial implications for DCM.

### Identification of Key Genes

**FIGURE 5:**
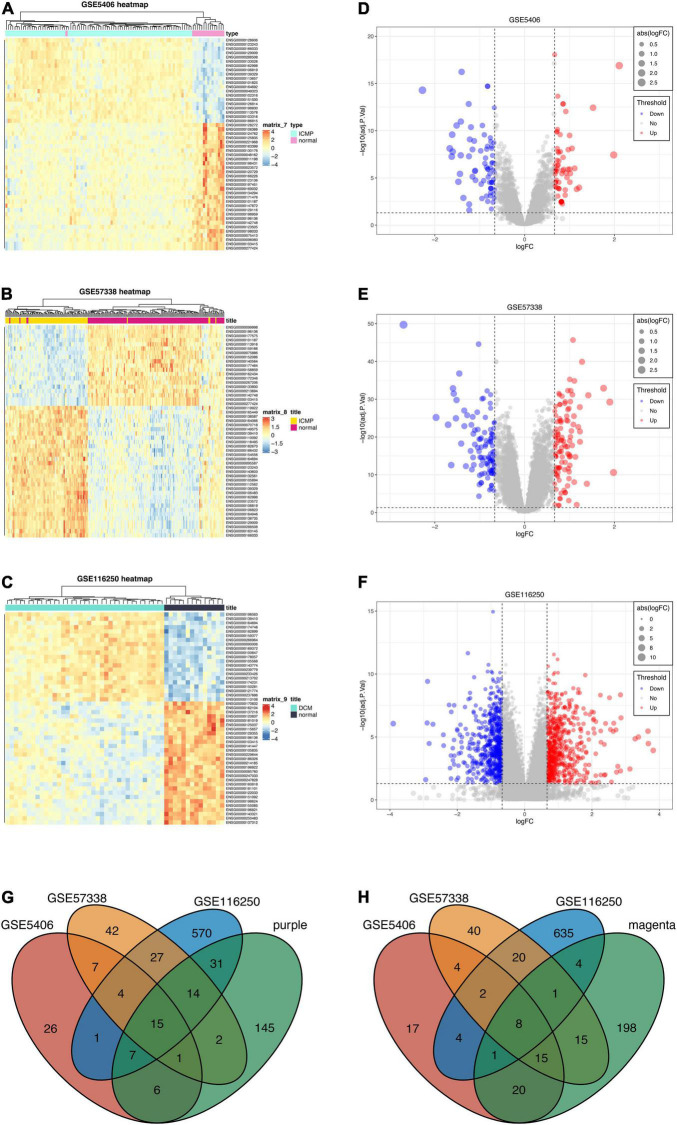
Differential expression analysis of three datasets and intersections for key genes. **(A–C)** Heatmaps of DEGs in three datasets (GSE5406, GSE57338, GSE116250). **(D–F)** Volcano plots of DEGs in three datasets (GSE5406, GSE57338, GSE116250). **(G)** The intersection between the up-regulated genes of three datasets and genes of the purple module. **(H)** The intersection between the down-regulated genes of three datasets and genes of the magenta module.

Three datasets (GSE5406, GSE57338, GSE116250) were enrolled in differential expression analysis, including 205 DCM samples and 174 controls. According to the filtering criteria (| log_2_(foldchange)| > 0.667 and *p*-value < 0.05), we obtained 67 up-regulated genes and 71 down-regulated genes from GSE5406 dataset. GSE57338 dataset has 112 up-regulated genes and 102 down-regulated genes. GSE116250 dataset has 669 up-regulated genes and 675 down-regulated genes. The result of the differential analysis was visualized in Figures 5A–F. Then, to identify the common DEGs of three datasets, we took intersections of up-regulated and down-regulated genes, respectively. The Venn diagram depicted the intersections (Figures 5G,H). As a result, there are 19 common up-regulated genes and 10 common down-regulated genes. Next, we filtered the key genes of WGCNA and differential analysis. A total of 23 key genes were identified, including fifteen key up-regulated genes and eight key down-regulated genes. All 23 genes were prepared for input variables of LASSO.

### Construction and Validation of Genetic Diagnosis Model

**FIGURE 6:**
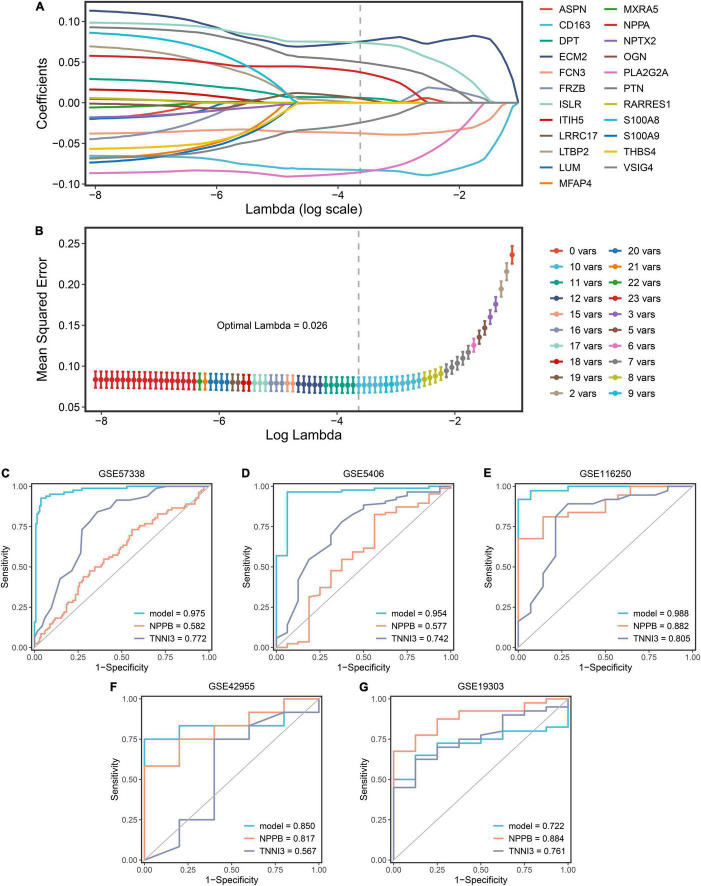
The construction and validation of the LASSO diagnostic model. **(A)** The processes of LASSO regression for screening variables and mapping each variable to a curve. **(B)** The log (λ) value was optimally selected by 10-fold cross-validation and plotted by the partial likelihood deviance. **(C–G)** The ROC curves of the LASSO model, NPPB, and TNNI3 in five datasets (GSE57338, GSE5406, GSE116250, GSE42955, GSE19303).

GSE57338 dataset was selected as the modeling dataset because of the largest sample size (82 DCMs and 136 controls). LASSO was applied to establish a diagnostic model using 23 genes previously mentioned. After analyzing, 10 genes had non-zero coefficients and were used for the final LASSO regression model (Figure 6A). Among these genes, six (PTN, ECM2, LRRC17, ISLR, DPT, NPPA) were upregulated in DCM while four (FCN3, VSIG4, CD163, PLA2G2A) were downregulated. The expression levels of 10 genes were validated in five datasets, which corresponded with our results basically (Supplementary Figure 1). The optimal λ was 0.026 (Figure 6B). The final model equation was: risk score = 0.376 + 0.050*PTN + 0.075*ECM2 + 0.005* LRRC17 + 0.074*ISLR—0.038*FCN3 + 0.006*DPT—0.024*VSIG4 + 0.038*NPPA—0.083*CD163—0.085* PLA2G2A. To validate the 10-gene diagnostic model, GSE5406, GSE19303, GSE42955, and GSE116250 were adopted as validation datasets. Then, plus the modeling dataset, a total of five ROC plots were displayed with AUCs (GSE57338: 0.975; GSE5406: 0.954; GSE116250: 0.988; GSE42955: 0.850; GSE19303: 0.722). To analyze the model’s superiority, we compared 10 genes with NPPB and TNNI3, genes of BNP and troponin, respectively (Figures 6C–G). The LASSO model performed significantly better than clinical biomarkers in most cohorts, representing that a relatively ideal diagnostic model was obtained.

### Prediction of Potential Pathways With Gene Set Enrichment Analysis

**FIGURE 7:**
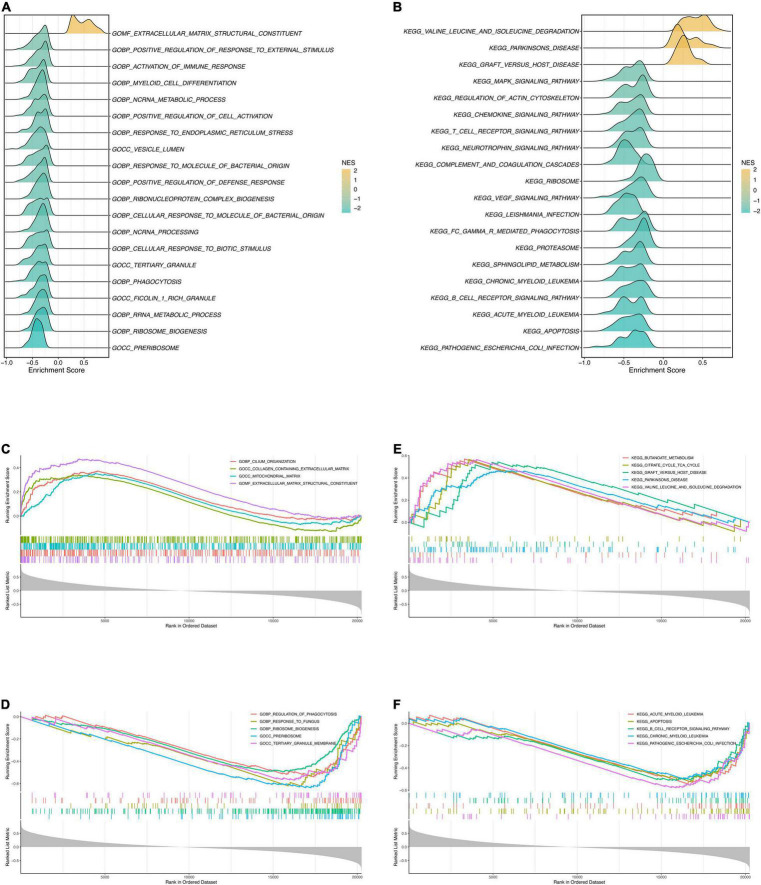
Gene Set Enrichment Analysis. **(A)** The ridge plot of the top 20 GO terms with ranked genes of the modeling dataset. **(B)** The ridge plot of the top 20 KEGG pathways with ranked genes of the modeling dataset. **(C,D)** The positive and negative top 5 GO terms with ranked genes of the modeling dataset. **(E,F)** The positive and negative top 5 KEGG pathways with ranked genes of the modeling dataset.

Prior to GSEA, the risk score and gene expression correlations were calculated and used for ranking genes. Then we conducted GSEA to explore the potential pathways of DCM. The most significant GO terms and KEGG pathways were exhibited in Figures 7A,B. Four positively correlated GO terms were enriched (Figure 7C), including “extracellular matrix structural constituent,” “cilium organization,” “mitochondrial matrix” and “collagen containing extracellular matrix.” Notably, terms about “extracellular matrix” were enriched once again, which is highly similar to the purple module’s functional enrichment. Arguably, the “extracellular matrix” is probably an essential pathway of DCM. The negative enrichment is shown in Figure 7D. On the other hand, the top 5 positive KEGG pathways were “valine leucine and isoleucine degradation,” “butanoate metabolism,” “Parkinson’s disease,” “graft vs. host disease,” and “citrate cycle TCA cycle” (Figure 7E). It was immune and molecule metabolism that genes were mainly enriched in. Relatively, the adverse pathways were “pathogenic escherichia coli infection,” “apoptosis,” “acute myeloid leukemia,” “B cell receptor signaling pathway,” and “chronic myeloid leukemia” (Figure 7F). So, we can rationally infer that DCM and myeloid leukemia may have common pathways.

### Immune Infiltration Analysis

**FIGURE 8:**
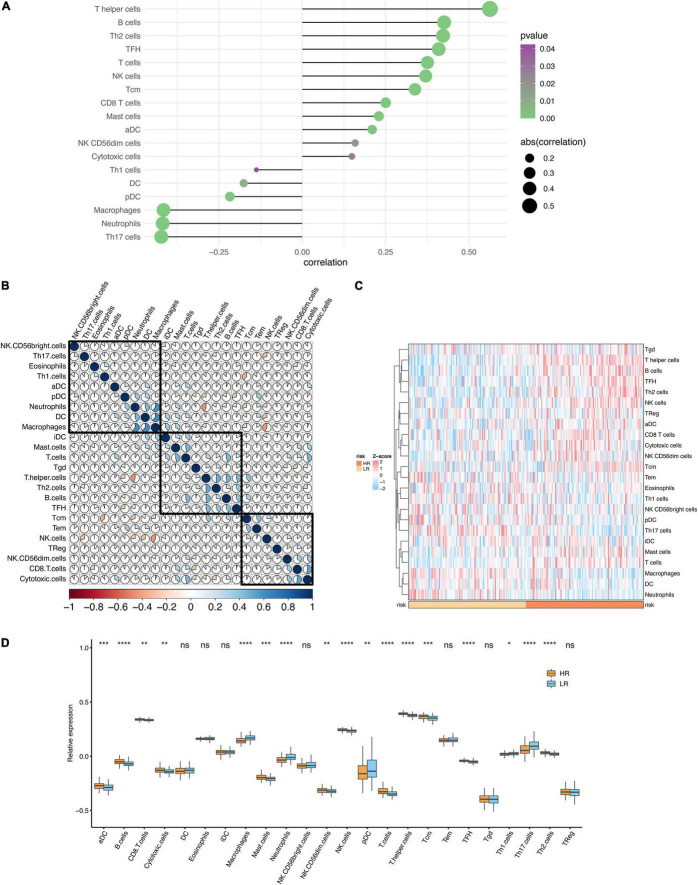
Immune infiltration analysis. **(A)** The lollipop plot of the correlation between the risk score and immune infiltration. **(B)** The heatmap of the correlations between different immune cells. **(C)** The heatmap of the immune infiltration in high and low-risk groups. **(D)** The boxplot of the immune infiltration in high and low-risk groups. *P < 0.05, **P < 0.01, ***P < 0.001. ****P < 0.0001; ns, no significance.

To gain insight into the immune infiltration of DCM, we used ssGSEA to calculate the immune cells abundance of the modeling dataset. Next, the risk score and immune cell abundance correlations were calculated (Figure 8A). It was noteworthy that T helper cells, B cells, and Th2 cells were significantly associated with DCM risk. The correlation of immune cells is plotted in Figure 8B. Additionally, the median risk score was used to classify the samples into high-risk and low-risk groups. Then we compared the immune infiltration of two groups (Figures 8C,D). The differences in immune infiltration were noticeable between high-risk and low-risk groups.

## Discussion

The pathogenesis of DCM remains unclear, resulting in non-specific treatments. Mechanical circulatory support and cardiac transplantation could probably prolong survival and reduce hospitalization in adults and children (19, 20). Hence, identifying potential genes and mechanisms of DCM is crucial for exploring new therapies and improving prognosis.

In our study, there were two modules that had high correlations with DCM, including the purple and the magenta module, which consists of 221 and 262 genes, respectively. GO terms about extracellular matrix (ECM) and focal adhesion were mainly enriched in the purple module. The maladaptive remodeling of ECM usually contributes to heart failure, including abnormal ECM degradation and immoderate ECM deposition. Then, the systolic and diastolic function of the heart would be impaired by these alterations (21). Similarly, the ECM-receptor interaction was also a significant term in KEGG pathways. The ECM cues were transmitted to intracellular signaling pathways by integrin, which can regulate cell apoptosis and movement. The reduction of integrin leads to ventricular dilatation and failure (22). It should be noted that the decrease of focal adhesion kinase (FAK) influences the intact function of integrin in DCM (23). Therefore, the genes in the “focal adhesion” pathway also play an essential role in the pathogenesis of DCM. Consequently, pathways related to the extracellular matrix may become potential intervention targets.

The magenta module was strongly negatively correlated with DCM. Genes in the magenta module were primarily enriched in the GO terms, such as “response to lipopolysaccharide,” “response to molecule of bacterial origin,” “response to oxidative stress.” It has been revealed that inflammation and oxidative stress are conducive to the development of DCM (24). Inflammation was often caused by bacterial infection. Notably, lipopolysaccharide (LPS) is a component of the outer wall in bacterial cell walls, exhibiting various biological activities when it acts on human cells (25). In addition, as one of the endotoxins, LPS can induce an inflammatory response by multiple cytokines, such as IL-6 (26). Oxidative stress plays a part in the process of heart failure, especially in DCM (27). Researchers found there was usually an increase of oxidative stress in the failing myocardium, which was likely to impact ventricular function in patients with DCM (28). On the other hand, there were several prominent enrichment of KEGG pathways of the magenta module, such as “IL-17 signaling pathway,” “Human T-cell leukemia virus 1 infection,” “Lipid and atherosclerosis.” These pathways were related to inflammation and immune. Studies have confirmed that IL-17 participates in cardiac remodeling induced by inflammation in post-myocarditis, resulting in DCM progression. IL-17 signaling pathway relies on T helper cells greatly. Researchers found that γδ T cells releasing IL-17 were the main T-cell population observed in the cardiomyopathy samples of mice (29). Studies also showed that IL-17A, mainly released by Th17 cells, plays a critical role in the progression of DCM and cardiac remodeling in mice. IL-17 may lead to the heart-specific upregulation of IL-6, TNFalpha, and IL-1beta and the recruitment of CD11b (+) monocyte and Gr1(+) granulocyte populations into the heart (30, 31). For this reason, treatment of anti-IL-17 monoclonal antibody has been applied in mice with myocarditis and gained a desirable efficacy of abrogating cardiac fibrosis and slowing down the aggravation of ventricular function (30). Since that, we can rationally assume that anti-IL-17 therapy might be a novel thought for patients with DCM.

In this study, the novel genes were obtained from the intersection of WGCNA and DEA, including 23 genes. Huang et al. performed DEA and identified some hub genes of DCM using GSE5406 dataset, which still differed crucially from our study (32). Our study enrolled four more datasets and used a more robust method to filter the DEGs, which was the combination with WGCNA. Hence, the latent mechanisms revealed in our study were more convincing and powerful. Furthermore, we employed a bunch of methods to gain an insight into DCM, making our study more comprehensive. Compared to other studies using WGCNA or DEA alone to identify genes (33, 34), the genes screened from two methods combined were more persuasive and valuable. These genes were not only from the key modules highly associated with DCM but also had differential expression between DCM samples and controls, which were more likely to be biomarkers in the foreseen future. Twenty-three key genes were used for dimensional reduction to build a gene diagnostic model. The classical LASSO was chosen from various machine-learning algorithms because of its excellent performance. Based on the optimal λ, an ideal diagnostic model was established by 10 genes. Except for the modeling dataset (GSE57338), another four were utilized as validation datasets to make the model more reliable and rigorous. Reassuringly, all AUCs were greater than 0.7, ranging from 0.722 to 0.988, which were completely acceptable. Huang et al. used LASSO to establish a prediction model of heart failure in DCM patients (32). To validate the value of genes in the prediction model, we compared it with our model in several datasets containing 12 genes of the model. Consequently, our diagnostic model showed a superior ROC significantly in two datasets (Supplementary Figure 1). Given the superb performance against the clinical biomarkers and other studies, our model may provide new diagnostic ideas to improve clinical practice.

We applied GSEA in exploring the critical mechanisms of DCM. Surprisingly, terms about extracellular matrix (ECM) still took a leading role, compared to the result mentioned previously. Apart from this, the mitochondrial matrix was also critical for DCM. Gene mutations could lead to mitochondrial alterations and worsen DCM. For instance, the most common genetic cause of DCM, truncating titin (TTN) variants, lead to pronounced mitochondrial dysfunction with increased ventricular arrhythmias, which are the lethal causes of DCM (35). KEGG pathways were mainly about metabolism. The citric acid cycle is a basic metabolism, playing a vital role in multiple metabolic pathways. Furthermore, Haas et al. (37) found that several metabolites connected with the citric acid cycle were significantly up-regulated with 5.7-fold in DCM. It is known that the citric acid cycle takes place in the mitochondrial matrix. Therefore, the mitochondrial matrix is inextricably linked to the metabolic pathways obtained from KEGG analysis. Although Parkinson’s Disease is hard to associate with DCM, these two diseases are indeed related. Regardless of the epidemiological linkages between them, common underlying mechanisms were proposed by Bhandari et al. (37). Parkin deficiency can result in the disruption of mitochondria. Then, the disrupted and normal mitochondria fuse, exacerbating DCM. According to this, the sharing pathways of Parkinson’s Disease and DCM probably turn out to be rational therapeutic targets, which will benefit the patients of two intractable diseases. As we assumed, there is indeed an association between leukemia and DCM. By regulating H3K4me2, mixed lineage leukemia 3 (MLL3) might impact the pathological process of DCM, which is a member of MLL families. With the increase of MLL3 expression, the H3K4me2 also elevated in the DCM hearts (38). Even if the evidence is still lacking to demonstrate the relationship between acute or chronic myeloid leukemia, studies exploring the linkage are promising.

Immune infiltration analysis revealed apparent differences between the high-risk and low-risk groups according to the lollipop plot, boxplot, and heatmap. T helper cells, B cells, and Th2 cells were significantly correlated with the risk of DCM, according to the analysis. These immune cells, as we know, are primarily involved in humoral immunity. Humoral immunity impacts DCM by numerous autoantibodies against cardiac cell proteins (39). However, immunoadsorption therapy resists the impaction by removing active autoantibodies from plasma. Besides, a significant increase of Th2 cells was observed in DCM patients compared with healthy volunteers, which is highly consistent with our results (40). Macrophages usually play a critical role in myocarditis. However, in our analysis, macrophages showed a negative correlation with DCM. In mice with dilated cardiomyopathy, reduction of CCR2- macrophages increased mortality and hindered ventricular remodeling and coronary angiogenesis, adaptive processes required to sustain cardiac output in the face of diminished cardiac contractility, according to a recent research (41). Another study found that self-renewing resident cardiac macrophages help to prevent unfavorable remodeling after myocardial infarction (42). Consequently, immunotherapy may exert a significant influence among specific patients with DCM.

## Limitations

We acknowledge some limitations in this study. First, all initial data were downloaded from the GEO database, lacking our own clinical data. Second, the keywords “dilated cardiomyopathy” and “DCM” could hardly cover all the investigations of DCM, which were suitable for our study. Thirdly, a sudden reduction of DEGs occurs when we added GSE42955 and GSE19303 into the differential analysis. In order to preserve the valuable genes for an ideal LASSO model, we decided to use three datasets to conduct differential analysis. Besides, DEGs from three datasets were taken intersections with the genes in the key modules of WGCNA, which made our key genes more robust and convincible. To relieve the concern about the left two datasets (GSE42955 and GSE19303), they were employed as validation cohorts to check the performance of the diagnostic model, which showed acceptable AUCs as well.

## Conclusion

In conclusion, this study identified 23 key genes and several crucial pathways of DCM using combined bioinformatic methods, which may inspire researchers to investigate further. We also constructed a 10-gene diagnostic model, offering a novel tool for diagnosing DCM in clinical practice.

## Data Availability Statement

The datasets presented in this study can be found in online repositories. The names of the repository/repositories and accession number(s) can be found in the article/Supplementary Material.

## Author Contributions

YZ and ZL designed the study. YZ, XY, SW, CG, and ZX integrated and analyzed the data. YZ, LL, LW, and HX wrote the manuscript. YZ, ZL, XY, and QD edited and revised the manuscript. All authors approved this manuscript.

## Conflict of Interest

The authors declare that the research was conducted in the absence of any commercial or financial relationships that could be construed as a potential conflict of interest.

## Publisher’s Note

All claims expressed in this article are solely those of the authors and do not necessarily represent those of their affiliated organizations, or those of the publisher, the editors and the reviewers. Any product that may be evaluated in this article, or claim that may be made by its manufacturer, is not guaranteed or endorsed by the publisher.

## Footnotes

1
http://www.ncbi.nlm.nih.gov/geo/
