# Supplementary material for: Exploring Key Genes to Construct a Diagnosis Model of Dilated Cardiomyopathy
Source: Front Cardiovasc Med. 2022 Apr 27;9:865096. doi: 10.3389/fcvm.2022.865096 (PMC9091505; doi:10.3389/fcvm.2022.865096)
Supplement: Supplementary file 2 [file Image_1.pdf]

Supplemental Figure

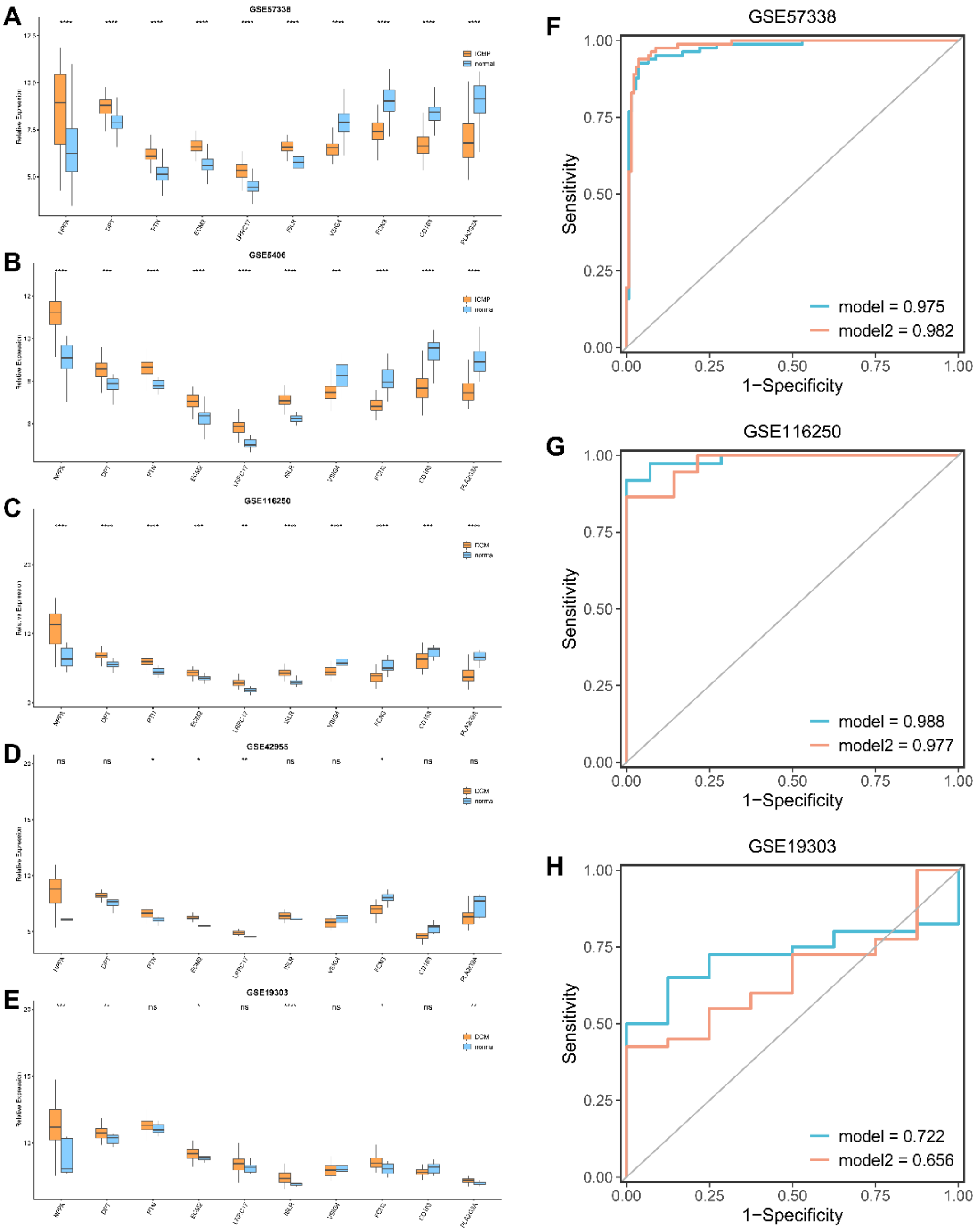

Figure S1. The validation of genes expression; The comparison with the other model. (**A-E**) Boxplots of model genes' expression level in five datasets. (**F-H**) The ROC curves of our model and the other model (model 2) in three datasets.
